# Effect of Magnetite Nanoparticles on Formation Damage Mitigation in Carbonate Rocks

**DOI:** 10.1021/acsomega.5c04774

**Published:** 2025-12-10

**Authors:** Ernani Dias da Silva Filho, Gregory Vinicius Bezerra de Oliveira, Fernanda Larissa do Vale Barbosa, Aeryslânnia Moreira da Nóbrega, Sérgio Taveira de Camargo Júnior, Vanessa Cristina Santanna, Marcos Allyson Felipe Rodrigues

**Affiliations:** † Department of Petroleum Engineering, 28123 Federal University of Rio Grande do Norte, 59064-970 Natal, Brazil; ‡ Department of Chemical Engineering, 28123 Federal University of Rio Grande do Norte, 59072-970 Natal, Brazil; § Petróleo Brasileiro S.A. − PETROBRAS, 21941-915 Rio de Janeiro, Brazil

## Abstract

Nanoparticles have shown a potential to reduce formation damage and enhance drilling efficiency, though return permeability data remain limited in the literature. This study explores the incorporation of Fe_3_O_4_ nanoparticles into water-based drilling fluids as a strategy to reduce formation damage in carbonate reservoirs. Nanoparticles were synthesized and added to the base fluid at concentrations of 0.25, 0.5, and 1.0% (w/v). Comprehensive physicochemical and rheological characterizations were performed, followed by return permeability tests using Indiana Limestone cores under simulated reservoir conditions. The results demonstrated that the addition of 0.25% Fe_3_O_4_ significantly enhanced return permeability (83.2%) compared to the base fluid (70.7%) and reduced the flow initiation pressure from 10.7 to 6.1 psi. Despite an increase in filtrate volume at higher concentrations, the 0.25% formulation exhibited the optimal trade-off between filtration control (2.5 mL), filter cake quality, and return permeability. Statistical evaluation confirmed the significance of the observed improvements (*p* < 0.05). These findings demonstrate the potential of Fe_3_O_4_ nanoparticles to enhance drilling fluid performance and reduce reservoir damage.

## Graphical abstract

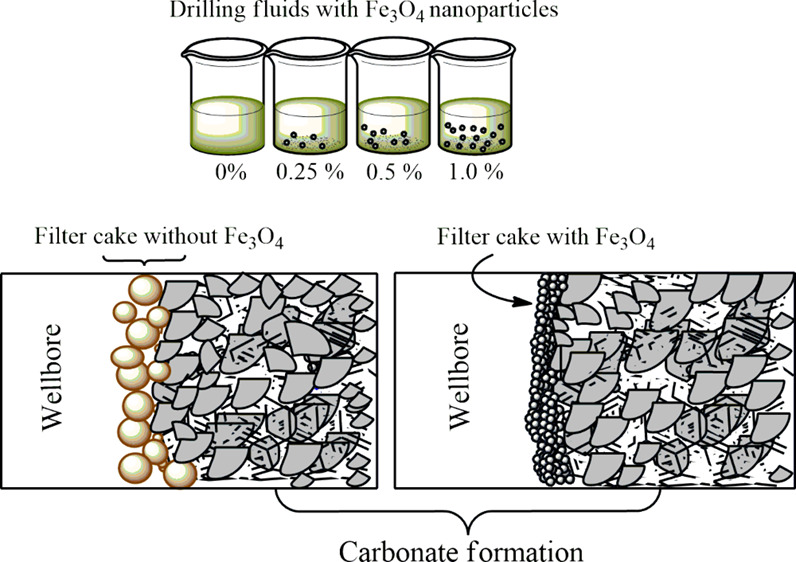

## 1 Introduction

The exploration and development of new drilling fluids are essential for the oil and gas industry, emphasizing novel formulations that adapt to the specific characteristics of geological formations. The main objective is to reduce formation damage, defined as any unintentional impairment to the flow of fluids into or out of a well. In other words, it results from the reduced ability of the rock to allow fluid flow in the near-wellbore region.^1,2^

The incorporation of nanoparticles into drilling fluids is a growing area of research. Although nanoparticles are still under development and remain minimally used, their application has attracted growing attention from researchers and industry to enhance the performance and efficiency of drilling fluids. Their use offers key advantages, such as reducing unfavorable wellbore conditions and improving the thermal stability of the fluid,^3^ while also minimizing formation damage and preventing fluid invasion into the reservoir.^3–5^

However, there is a consensus that the laws governing behavior at the nanoscale significantly differ from those that apply at larger scales.^6^ There is still no satisfactory conclusion regarding the relationship between nanoparticle size, surface charge, nature, concentration, and their impact on filtrate volume.^7^

Recent advances in nanotechnology have highlighted the potential of nanoparticles to improve drilling fluid performance, particularly in terms of filtration control, thermal stability, and mitigation of formation damage. A wide variety of nanoparticles has been investigated, including silica (SiO_2_), titanium dioxide (TiO_2_), alumina (Al_2_O_3_), and carbon-based nanomaterials such as graphene oxide. These materials have shown beneficial effects on rheological properties, fluid loss reduction, and filter cake quality. However, challenges such as colloidal stability, particle aggregation at higher concentrations, cost-effectiveness, and environmental compatibility remain critical barriers to large-scale application.^8–10^ In this context, Fe_3_O_4_ nanoparticles offer distinctive advantages compared to other nanomaterials. Due to their superparamagnetic nature, they present strong colloidal stability under alkaline conditions, the possibility of magnetic recovery, and the ability to form denser and thinner filter cakes, thereby minimizing solid invasion into the porous matrix. Furthermore, magnetite nanoparticles are relatively low-cost and abundant compared to functionalized silica or carbon-based nanoparticles, which makes them an attractive and sustainable alternative for drilling fluid formulations.^11,12^ Despite these advantages, systematic studies on the relationship between Fe_3_O_4_ concentration, filtration volume, and return permeability in carbonate formations remain scarce, which highlights the novelty and relevance of the present work.

Ibrahim and collaborators studied the influence of zeta potential in silicon dioxide (SiO_2_) nanoparticles, with an average size of 30 nm, on filtrate volume and its effect on flow permeability in porous media. Their experiments were conducted using Berea sandstone cores under a confining pressure of 3,000 psi, a pore pressure of 1000 psi, an overbalance of 500 psi, a flow rate of 0.5 mL/min, and a temperature of 60 °C. The results indicated that the addition of unfunctionalized SiO_2_, SiO_2_ functionalized with a low carboxyl (−COOH) content, and SiO_2_ functionalized with a high carboxyl content reduced filtration by 13, 33, and 36%, respectively. Regarding the return permeability of the rock after exposure to drilling fluids without nanoparticles and with unfunctionalized SiO_2_, a reduction of 47 and 82% was observed, respectively. However, exposure to drilling fluids containing SiO_2_ functionalized with low and high carboxyl content resulted in reductions of only 21 and 15%, respectively. These results highlight the importance of nanoparticle surface charge in enhancing return permeability in porous media.^7^

In our study, three types of water-based drilling fluids containing Fe_3_O_4_ nanoparticles smaller than 14 nm were developed at concentrations of 0.25, 0.5, and 1.0% (w/v). The main objective was to investigate the potential of improving drilling fluid performance by adding nanoparticles as additives, aiming to enhance return permeability after fluid circulation in carbonate rocks such as Indiana Limestone. The nanoparticles were synthesized and subsequently incorporated into the drilling fluid. Physicochemical analyses included measurements of weight, rheological parameters, filtration, solids content, alkalinity, chloride content, and pH. Return permeability tests were conducted under the following conditions: confining pressure was maintained at 3000 psi, and the backpressure was set to 1000 psi. The tests were conducted at room temperature (25 °C), and an overbalance of 300 psi was applied. A 4% potassium chloride (KCl) brine was used as the return fluid, while the drilling fluid was formulated according to the specifications provided by Petrobras/Brazil. These parameters were selected to reflect conditions similar to those encountered in real drilling and production operations, thereby ensuring the representativeness and applicability of the return permeability test results.

## 2 Experimental Section

In the procedures applied during the experimental analyses, ferrous sulfate heptahydrate (FeSO_4_·7H_2_O, PA, Vetec, Brazil), ferric chloride hexahydrate (FeCl_3_·6H_2_O, PA, Vetec, Brazil), and sodium hydroxide (NaOH, PA, Dinâmica, Brazil) were used as received. Distilled water was also used in all experiments.

The drilling fluids were formulated by adding viscosifying agents, polymers, weighting agents, buffering agents, and biocidal agents, complemented by the inclusion of Fe_3_O_4_ nanoparticles, which constitute the focus of this research.

### 2.1 Preparation of Fe_3_O_4_ Nanoparticles

Fe_3_O_4_ nanoparticles were obtained by the coprecipitation method, using the following procedures:

1. The experimental procedure consists of dissolving 0.01 mol of Fe^2^^+^ in 5 mL of distilled water in a beaker, referred to as “beaker A.” Then, in beaker B, 0.02 mol of Fe^3^^+^ is dissolved in 5 mL of distilled water. Subsequently, the solutions from beakers A and B are mixed until a homogeneous mixture is obtained.
2. Next, in a beaker designated as “beaker C,” an 8 mol·L^–1^ NaOH solution was prepared and added dropwise (∼1 mL·min^–1^) to the Fe^2^^+^/Fe^3^^+^ mixture under vigorous mechanical stirring (≈300 rpm) at room temperature (25 °C). Stirring was maintained for 30 min to ensure homogeneity and nanoparticle growth, and magnetite was immediately formed according to the coprecipitation reaction (eq 1). These conditions are consistent with those typically employed in magnetite synthesis by coprecipitation.^13^
$${{\mathrm{Fe}}^{2+}}_{\left(\mathrm{aq}\right)}+2{{\mathrm{Fe}}^{3+}}_{\left(\mathrm{aq}\right)}+8{{\mathrm{OH}}^{-}}_{\left(\mathrm{aq}\right)}\to {\mathrm{Fe}}_{3}O_{4(s)}+4H_{2}O_{\left(l\right)}$$ 1
3. The nanoparticles formed were then washed with distilled water until the water’s pH reached 9.0, corresponding to the drilling fluid’s pH.

### 2.2 Drilling Fluid Formulation with Nanoparticles

The Brazilian oil company Petrobras (Brazil) provided the drilling fluid and underwent complete physicochemical characterization. The composition of the base drilling mud used in this research is presented in Table 1.

**Table 1:** Composition of Base Drilling Mud

product	concentration (lb/bbl)
industrial water	331.2
NaCl	15.8
inorganic salt 2	1.5
viscosifier 1	1.2
viscosifier 2	8
defoamer	0.3
alkalizing agent	1
barite	19
antibacterial agent	0.19

Following the characterization of the base drilling mud, three drilling fluids were formulated with nanoparticles at 0.25, 0.5, and 1.0% (w/v) concentrations. A new physicochemical characterization was then performed to assess any influence on the fluid’s parameters. These concentrations were selected to minimize alterations to the original fluid behavior and to ensure a low-cost, low-complexity approach while covering a representative range for performance evaluation.

To minimize nanoparticle aggregation and ensure adequate dispersion in the drilling fluids, the Fe_3_O_4_ nanoparticles were incorporated under high-shear mechanical stirring, and the fluid pH was adjusted to approximately 9.0 to promote colloidal stability. This pH value is also consistent with the natural range of the base fluid, thus avoiding significant deviations from its original formulation.

### 2.3 Characterization

The laboratory experiments were conducted to comprehensively characterize drilling fluid formulations, incorporating nanoparticles, focusing on data for evaluating postcirculation permeability in carbonate rocks. The characterization involved various fundamental tests, including determining viscosity, pH, density, permeability, filter retention, solids content, salinity, and all necessary chemical analyses for drilling fluid characterization, including the incorporation of Fe_3_O_4_ nanoparticles.

The characterization procedure begins with preparing the fluid, which involves the controlled addition of specific additives corresponding to each formulation, including the nanoparticles, into distilled water. This step is carried out under mechanical stirring using a Hamilton Beach mixer, with timed mixing intervals for each additive. After formulation, the fluid rests for 30 min before undergoing rheological analysis. A small sample of 50 mL is also collected and left to rest for the same period to measure the pH.

For rheological analysis, the fluid is transferred to the container of a Fann 35A viscometer, where rotational torque readings are recorded across a range from 600 to 3 rpm. These readings are influenced by fluid viscosity, rotor speed, temperature, and pressure environmental conditions. After mathematical treatment of the collected data, rheological parameters are determined, including initial gel strength (G_i_) and final gel strength (G_f_), plastic viscosity (PV), apparent viscosity (AV), and yield point (YP). Subsequently, the specific gravity (SG) was measured using a densimeter (pressurized liquid balance) to determine the density of a fluid sample, where pressurization reduces the likelihood of measurement distortion caused by the presence of gas and bubbles.

The filtrate volume and filter cake thickness were determined through the low-pressure, low-temperature (LPLT) filtration test, in which a specific volume of drilling fluid was subjected to a pressure of 100 psi. Over 30 min, the filter paper with the formed cake was collected, and the filtrate volume was recorded. Using this collected filtrate volume, the salinity of the fluid was determined for each 1 mL sample by titration with a silver nitrate (AgNO_3_) solution, where silver nitrate reacts with chloride ions (Cl^–^) present in the samples, forming a silver chloride (AgCl) precipitate. Similarly, alkalinity was determined by titration using 1 mL samples of the drilling fluid, in which titration was carried out with a standardized hydrochloric acid (HCl) solution. The amount of acid consumed in the titration was used to calculate the alkalinity of the fluid.

The solids content test was conducted using a 10 mL sample of the drilling fluid subjected to high temperatures in a retort. During the test, the sample was heated, causing the volatile components of the fluid to evaporate, while suspended solids and other nonvolatile residues were retained. The volume of the resulting condensate was then measured in milliliters (mL), and the percentage of solids present in the drilling fluid was determined through appropriate mathematical analysis. This procedure enables an accurate assessment of the solids content in the fluid, providing valuable information for the control and optimization of the drilling fluid formulation.

#### 2.3.1 X-Ray Diffraction (XRD)

The X-ray diffractogram was obtained using a Bruker D2 Phaser benchtop diffractometer, manufactured in Germany. The samples were analyzed over a Bragg–Brentano geometric angle range (θ–2θ) from 10° to 80°, with a step size of 0.02° and a scanning rate of 3°/min. Cu Kα radiation (λ = 1.54 Å) was used. The analyses were conducted at room temperature (25 °C). The XRD results were interpreted using the ICSD database and refined using the Rietveld method.^14^

#### 2.3.2 Transmission Electron Microscopy (TEM)

Morphological characterization of the Fe_3_O_4_ nanoparticles was conducted using a JEM-1011 transmission electron microscope operated at 100 kV in bright-field mode. The resulting images were used to evaluate particle shape and dispersion.

#### 2.3.3 Vibrating Sample Magnetometry (VSM)

Magnetic behavior data were acquired at 25 °C using a LakeShore vibrating sample magnetometer (VSM, model 7400, USA) with a maximum magnetic field amplitude of 15 kOe. Before the experiments, the system was calibrated using a pure nickel sample as a standard. Subsequently, after weighing the nanoparticle sample, the magnetic response was expressed in emu/g.

#### 2.3.4 Rock Characterization

Indiana Limestone cores, composed predominantly of calcite (>98%), were used in this study. The XRD analysis confirmed the dominance of calcite, while the small fraction of other components can be considered minor impurities not detected by XRD. The pore system is characterized mainly by intergranular macropores and secondary microporosity, features typical of this outcrop analogue. The plugs exhibited relatively homogeneous petrophysical properties, with porosity values ranging from 16.7% to 20.5% and permeability between 15.3 and 43.7 mD. These characteristics make Indiana Limestone a widely used standard in core flooding experiments for carbonate reservoirs.

For this study, core plugs of Indiana Limestone with dimensions of 1.5 inch in diameter and 3 in. in length were characterized to determine their basic petrophysical properties. Porosity was measured by applying Boyle’s law using helium gas in the HEP-E porosimeter (Vinci Technologies). Permeability was measured under steady-state conditions using nitrogen gas with the GPE-100 gas permeameter, also from Vinci Technologies. The procedure followed Darcy’s law and included a Klinkenberg correction to improve the accuracy of the results.^15^ This gas permeability data was used solely to select plugs with similar permeability levels. Liquid permeability was subsequently measured during flow experiments, and these values were used for calculating return permeability, as detailed in a subsequent section.

#### 2.3.5 Return Permeability Tests

Return permeability tests were conducted following the methodology described by Barbosa et al.,^16^ using the Formation Damage Core Flood System (FDS) manufactured by DCI Corporation. The experimental setup is illustrated in Figure 1.

**Figure 1:**
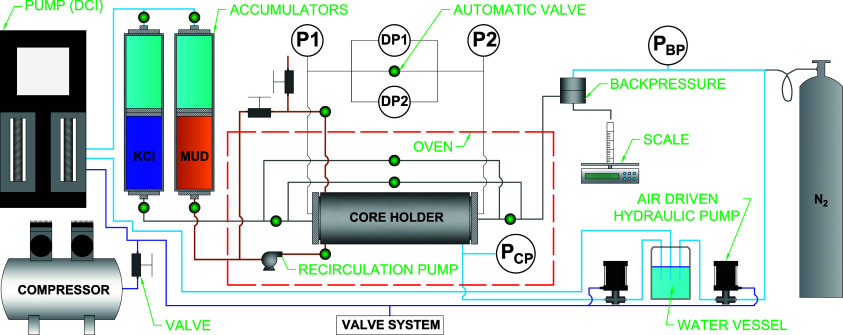
Experimental setup and process flowchart for formation damage tests. Reprinted with permission from Barbosa, F. L. V.; Bezerra de Oliveira, G. V.; Silva Filho, E. D.; et al. *Fuel*, 2026, 405, 136706. Copyright 2026 Elsevier.^16^

Under reservoir conditions, this equipment simulates the interaction between the rock and the drilling fluid, either in tangential flow direction (fluid circulating in the well during drilling) or axial flow direction (static well). The return permeability tests were conducted in four stages:

1. In this stage, the accumulators are filled with brine and drilling fluid. The drilling fluid is placed into the accumulator, which is equipped with an integrated agitation system that keeps the fluid homogenized throughout the entire test.
  The rock core (in this case, Indiana Limestone), previously saturated with brine, is inserted into the FDS core holder, and the confining fluid (distilled water) is used to fill the system. Once filled, the confining pressure (3000 psi) and backpressure (1000 psi) are applied.
2. In this stage, brine is injected into the core in the production direction at a 2 mL/min flow rate until steady-state flow conditions are achieved. This allows for the determination of the core’s absolute permeability.
3. After the determination of *k*_1_, the drilling fluid accumulator is pressurized until the overbalance pressure (300 psi) is reached. Then, the fluid comes into contact with the core face, circulating tangentially across the sample surface with the aid of the circulation pump in the system, allowing infiltration into the sample face. For this stage, the injection pump operates under constant pressure conditions to maintain the desired overbalance pressure for approximately 2 h.
4. After the filter cake formation, brine is once again injected from the production side at a 2 mL/min flow rate until steady-state flow conditions are achieved, to determine the permeability (*k*_2_).

## 3 Results and Discussion

### 3.1 Nanoparticle Characterization

#### 3.1.1 XRD

##### 3.1.2 Debye–Scherer Method

The structural characterization of the Fe_3_O_4_ sample was performed using X-ray diffraction (XRD), as shown in Figure 2. Rietveld refinement was carried out using the MAUD software,^17^ revealing that the Fe_3_O_4_ nanoparticles exhibit an inverse spinel structure [*Fd*3̅*m* (227), ICSD-26410], with characteristic peaks associated with magnetite planes, identified by 2θ values of 18.3 (111), 30.2 (220), 35.6 (311), 43.2 (400), 53.5 (422), 57.2 (511), and 62.8 (440).^13^ The crystallite size of the Fe_3_O_4_ nanoparticles was first estimated using the Debye–Scherrer equation, a classic approach commonly employed in X-ray diffraction (XRD) analysis. This method assumes that the broadening of diffraction peaks arises primarily from the crystals’ finite size. While it does not account for internal strain or defects, it provides a quick and useful approximation of the average crystallite dimension. The crystallite size was determined using the Debye–Scherrer equation (eq 2),^13^ resulting in a size of 11.8 nm.

$$d=\frac{K\lambda }{\beta \cos\;\theta }$$ 2

where *d* represents the average crystallite size, *K* is the shape factor, λ is the wavelength of the radiation, β is the full width at half-maximum (fwhm) of the most intense diffraction peak (in this case, corresponding to the (311) plane), and θ is the angle associated with that peak.

**Figure 2:**
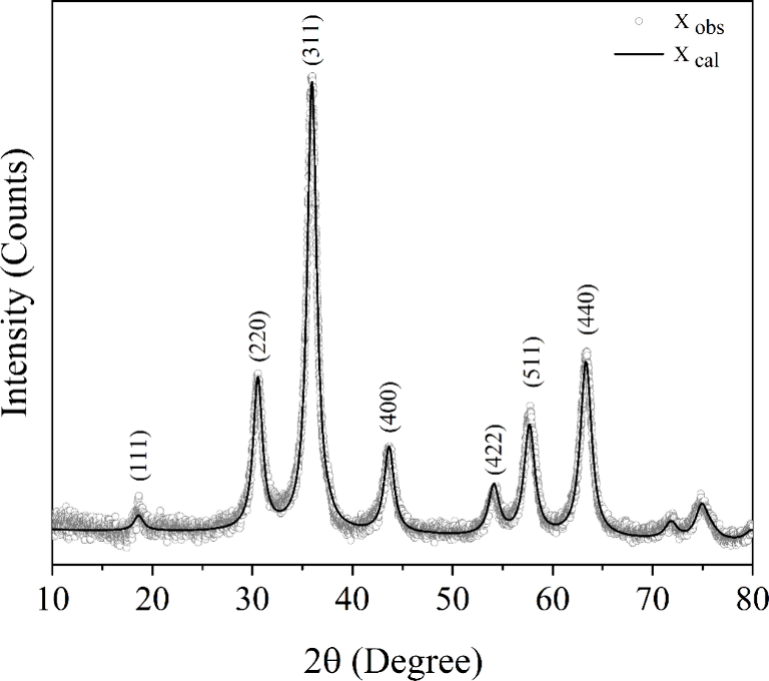
XRD patterns for the magnetite sample. The circles (gray) represent the experimental values, while the solid lines (black) correspond to the values calculated using the Rietveld method.

#### 3.1.3 Williamson–Hall method

To complement the crystallite size estimation and gain insights into the internal structural strain of the nanoparticles, additional analysis was performed using the Williamson–Hall (WH) method. Unlike the Scherrer equation, which assumes that peak broadening arises solely from finite crystal size, the WH method allows for separating two main sources of peak broadening in XRD: crystallite size and microstrain. This approach provides a more complete understanding of the nanoscale features of the synthesized Fe_3_O_4_ particles.^18^ The WH equation can be expressed as follows (eq 3):

$$\beta \cos\theta =\frac{K\lambda }{d}+4\varepsilon \sin\theta$$ 3

Here, ε represents the intrinsic strain in the nanoparticle structure.^19^ The Williamson–Hall plot (Figure 3) was obtained by plotting (β cos θ) versus (4 sin θ), resulting in a linear equation. The estimation of the average crystallite size was derived from the *y*-intercept, while the microstrain was calculated from the slope of the line obtained in the linear fit. The linear regression showed a coefficient of determination (*R* ^2^) of 0.97, indicating a good correlation with the experimental data. The estimated average crystallite size was 13.8 nm, and the microstrain was 0.026, which may be associated with surface disorder due to the small particle size.^20^

**Figure 3:**
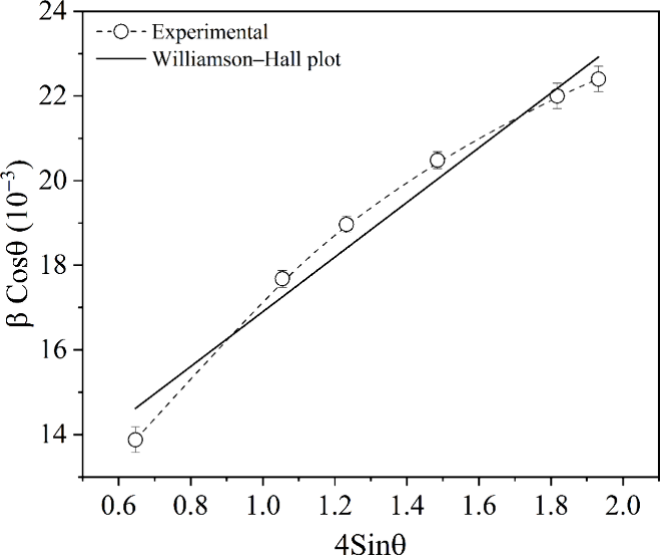
Williamson–Hall plot for magnetite.

#### 3.1.4 TEM

Figure 4 shows the transmission electron microscopy (TEM) image of the synthesized Fe_3_O_4_ nanoparticles and the corresponding particle size distribution histogram. The nanoparticles exhibit a predominantly spherical morphology with good dispersion. The histogram, generated using ImageJ software from a statistically relevant number of particles, indicates an average diameter of 10.1 ± 4.0 nm.

**Figure 4:**
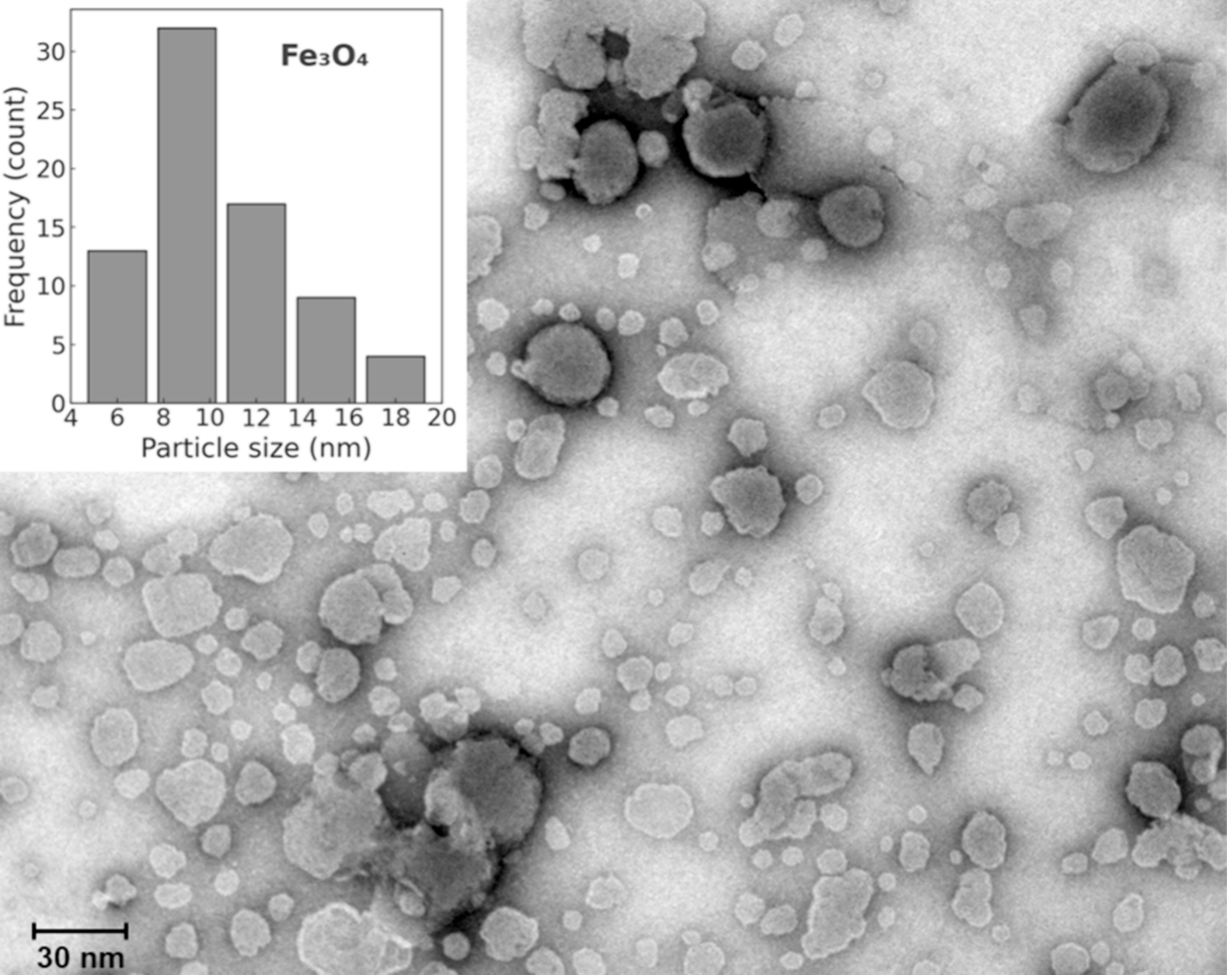
TEM image of magnetite nanoparticles and corresponding particle size distribution histogram.

These findings are consistent with the X-ray diffraction (XRD) analysis, which confirmed the crystalline structure characteristic of magnetite and indicated crystallite sizes of similar magnitude. The agreement between TEM and XRD results supports the successful synthesis of well-defined Fe_3_O_4_ nanoparticles and validates the reliability of the coprecipitation method used in this study.

#### 3.1.5 VSM

To confirm the nanoscale nature of the synthesized Fe_3_O_4_ particles, magnetic characterization was performed using vibrating sample magnetometry (VSM). This technique measures the magnetization of a material as a function of the applied magnetic field and is particularly useful for identifying superparamagnetic behavior commonly observed in particles smaller than ∼20 nm.^21^ The presence of such behavior serves as additional evidence of the nanoparticulate nature of the synthesized magnetite.

Figure 5 shows the magnetization curve for magnetite at a temperature of 25 °C. An S-shaped curve with no significant hysteresis indicates that the coercive field (*H*_c_) and remanent magnetization (*M*_r_) are nearly zero. Quantitatively, the saturation magnetization (*M*_s_) was 65.2 emu·g^–1^, and the coercive field values ranged from 0.01 to 0.04 kOe, while the remanent magnetization remained below three emu·g^–1^. These results are characteristic of superparamagnetic behavior, which is typically observed only in nanoparticles,^22–24^ and are consistent with the data obtained from the XRD analysis.

**Figure 5:**
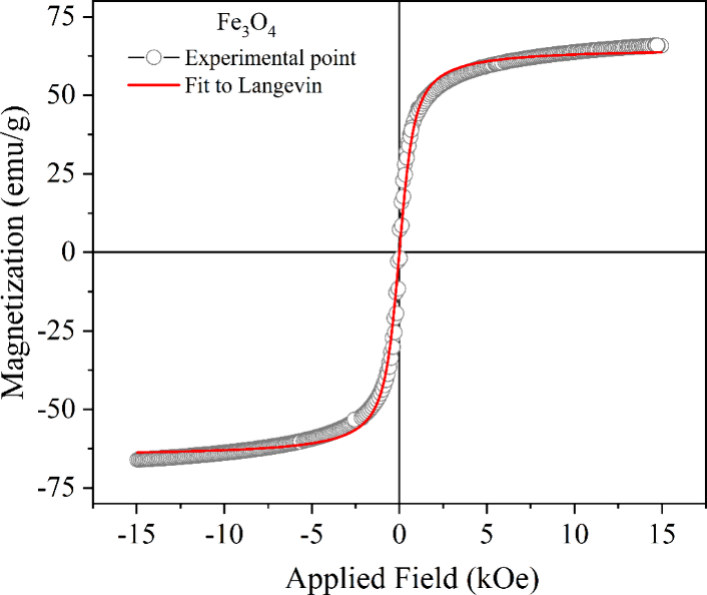
Magnetization curve of Fe_3_O_4_ nanoparticles at 25 °C. The red line represents the fit of the experimental magnetization to the Langevin saturation function.

The magnetization of Fe_3_O_4_ was also analyzed through the application of the Langevin equation to explain its superparamagnetic nature (eq 4). This equation, commonly used to describe the magnetic behavior of nanoparticles.

$$M=M_{s}L(\beta \mu H)=M_{s}\left[\coth(\beta \mu H)-\frac{1}{\beta \mu H}\right]$$ 4

where β = 1/*k*_B_ *T*. Here, *k*_B_ is the Boltzmann constant, *T* is the absolute temperature, *M*_s_ is the saturation magnetization of Fe_3_O_4_, and μ is the magnetic moment of the particle.^25,26^ The analysis of the experimental data through fitting the Langevin function showed excellent correlation, with a high coefficient of determination (*R* ^2^ = 0.998). These results indicate strong agreement between the theoretical model and the experimental results, thus confirming the superparamagnetic behavior of the studied material.^27,28^

### 3.2 Drilling Fluid Characterization

Table 2 presents the results of the analyses performed on the drilling fluid in the absence and presence of nanoparticles, as detailed in Section 2.3. This characterization was carried out to evaluate the influence of the nanoparticles in comparison with the original fluid, without the presence of these particles.

**Table 2:** Physicochemical and Rheological Properties of the Drilling Fluids with and without Fe_3_O_4_ Nanoparticles^a^

characterization	DF without Fe_3_O_4_	DF with 0.25% Fe_3_O_4_	DF with 0.5% Fe_3_O_4_	DF with 1.0% Fe_3_O_4_
plastic viscosity (cP)	20.50 ± 0.71	21.67 ± 0.47	21.67 ± 0.94	21.00 ± 1.63
apparent viscosity (cP)	36.08 ± 2.66	43.67 ± 1.25	43.33 ± 1.25	42.50 ± 2.48
yield point (100 lbf/ft^2^)	40.70 ± 0.94	44.00 ± 1.63	43.33 ± 0.94	43.00 ± 2.94
initial gel strength (100 lbf/ft^2^)	11.67 ± 0.94	12.00 ± 0.82	12.33 ± 0.47	13.00 ± 0.82
final gel strength (100 lbf/ft^2^)	14.67 ± 0.94	14.67 ± 0.47	14.67 ± 0.47	15.00 ± 0.94
pH	8.90 ± 0.22	9.50 ± 0.15	9.67 ± 0.31	9.32 ± 0.67
mud weight (lb/gal)	9.00 ± 0.00	9.00 ± 0.00	9.00 ± 0.00	9.00 ± 0.00
static API filtration (mL)	5.53 ± 0.09	4.93 ± 0.09	4.97 ± 0.09	5.20 ± 0.50
[NaCl] (mg/L)	46,200 ± 423	45,100 ± 500	45,100 ± 352	47,300 ± 507
filtrate alkalinity (mL)	1.6 ± 0.00	1.36 ± 0.09	1.30 ± 0.00	1.23 ± 0.05
solids content (%)	3.85 ± 0.50	5.32 ± 0.93	4.30 ± 0.59	3.89 ± 0.89
filter cake thickness (mm)	0.73 ± 0.02	0.54 ± 0.00	0.60 ± 0.05	0.56 ± 0.01

a DF: drilling fluid; [NaCl]: concentration; and API: American Petroleum Institute.

Based on the results, it was observed that the apparent viscosity of the fluid slightly increased with the presence of Fe_3_O_4_, indicating greater resistance to flow. This observation is supported by the increases in yield point, initial gel strength, and final gel strength, suggesting that the fluid had a higher load-bearing capacity and resistance to deformation.^29^ Similar improvements in rheological properties have been observed with other types of nanofluids, particularly those incorporating 2D nanomaterials, which demonstrate significant potential to enhance viscosity and yield point as the concentration of nanoparticles increases.^30^ For instance, Zamora-Ledezma et al. reported that adding graphene oxide (GO) to nanofluid formulations led to a 5-fold increase in viscosity and up to a 10-fold increase in yield stress at GO concentrations above 0.075 wt %, compared to a native XG-based formulation.^31^

Additionally, a slight increase in pH was observed with the addition of nanoparticles, while the mud weight remained stable. Static filtration and fluid salinity showed minimal variation with the different nanoparticle concentrations, indicating stability in these properties. The same applies to the filtrate volume; however, the variations are more noticeable in porous media flow tests, which will be discussed in Section 3.4. The NaCl concentration and solids content showed some fluctuations, but without a clear pattern associated with increasing nanoparticle concentration.

Regarding filter cake thickness, an ANOVA statistical analysis (*p* = 0.0004) revealed that the variations among the formulations are statistically significant. The formulation containing 0.25% Fe_3_O_4_ produced the thinnest and most uniform filter cake (0.54 ± 0.00 mm), indicating superior control over solid deposition and a greater ability to minimize formation damage. These findings demonstrate that the incorporation of Fe_3_O_4_ nanoparticles has a measurable and beneficial impact on the structure and performance of the filter cake.

### 3.3 Rock Characterization

The Indiana Limestone core plugs exhibited relatively homogeneous petrophysical properties, with liquid permeability values ranging from 15.26 to 43.72 mD and porosity between 16.71 and 20.52%. Most samples presented porosity values around 19%, with permeability generally decreasing as porosity decreased. These results confirm the suitability of the cores for flow experiments, given their intermediate permeability range and representative carbonate porosity.

### 3.4 Core Flooding Tests

Return permeability tests were conducted to assess the efficiency of using nanoparticles to reduce formation damage. Table 3 summarizes the parameters obtained from the tests performed with different nanoparticle concentrations: return permeability, filtrate volume, and flow initiation pressure (FIP).

**Table 3:** Parameters Obtained from Tests with and without Nanoparticles

nanoparticle concentration (%)	return permeability (%)	filtrate volume (mL)	FIP (psi)
0	70.7 ± 3.0	2.0 ± 0.2	10.7 ± 1.1
0.25	83.2 ± 2.4	2.5 ± 0.2	6.1 ± 2.5
0.5	81.3 ± 3.3	3.0 ± 0.5	7.6 ± 0.5
1.0	78.5 ± 3.1	3.3 ± 0.3	8.8 ± 1.4

The return permeability tests demonstrated a clear improvement in core permeability after circulation with fluids containing Fe_3_O_4_ nanoparticles, particularly at a concentration of 0.25%, which yielded a recovery of 83.2% compared to 70.7% for the base fluid. This improvement can be attributed to the ability of nanoparticles to reduce internal filter cake formation and stabilize the mudcake structure, thereby minimizing pore blockage and formation damage. These benefits are consistent with those reported by,^32^ who highlighted that nanoparticles contribute to the reduction of formation damage, control of fluid loss and filter cake thickness, as well as improvements in heat transfer, lubrication, and rheological fluid properties, such as viscosity.

Medhi et al.^10^ also reviewed the role of iron oxide nanoparticles in drilling fluids, highlighting their effectiveness in enhancing fluid stability and minimizing filtrate penetration into the formation, which supports these observations regarding performance improvement in porous media flow conditions. Similarly, Gokapai et al.^33^ reported that the inclusion of nanoparticles in drilling fluids can significantly reduce fluid loss and improve filter cake quality, which contributes to the protection of formation permeability. These mechanisms are consistent with the observed reduction in flow initiation pressure and the superior return permeability recorded for the 0.25% Fe_3_O_4_ formulation in this study. The reduced efficiency observed at higher Fe_3_O_4_ concentrations may be attributed to nanoparticle agglomeration, which can impair dispersion and increase flow resistance. This trend is further illustrated by the sedimentation test images (Figure 6) based on Rocha et al.^34^ Immediately after preparation (0 h) and after 1 and 2 h of rest under ambient conditions, no visible sedimentation or large aggregates were observed in the 0.25, 0.5, or 1.0% (w/v) suspensions, indicating that the Fe_3_O_4_ nanoparticles remained relatively stable in static conditions. However, the 1.0% suspension exhibited a darker coloration compared to the lower concentrations, reflecting its higher concentration of dispersed particles. Unlike other oxide nanofluids reported in the literature, no clear sediment layer was detected within 2 h, which suggests that agglomeration was not macroscopically evident in these samples. Nevertheless, it is important to note that such static laboratory tests do not replicate the dynamic flow and pressurized environment of core flooding experiments. Under those conditions, nanoparticle aggregation may still occur, which could explain the reduced return permeability observed at higher concentrations. Because of their high surface-to-volume ratio, nanoparticles have a natural tendency to minimize surface energy through agglomeration, and at higher loadings this effect may contribute to the decline in return permeability observed in this study.

**Figure 6:**
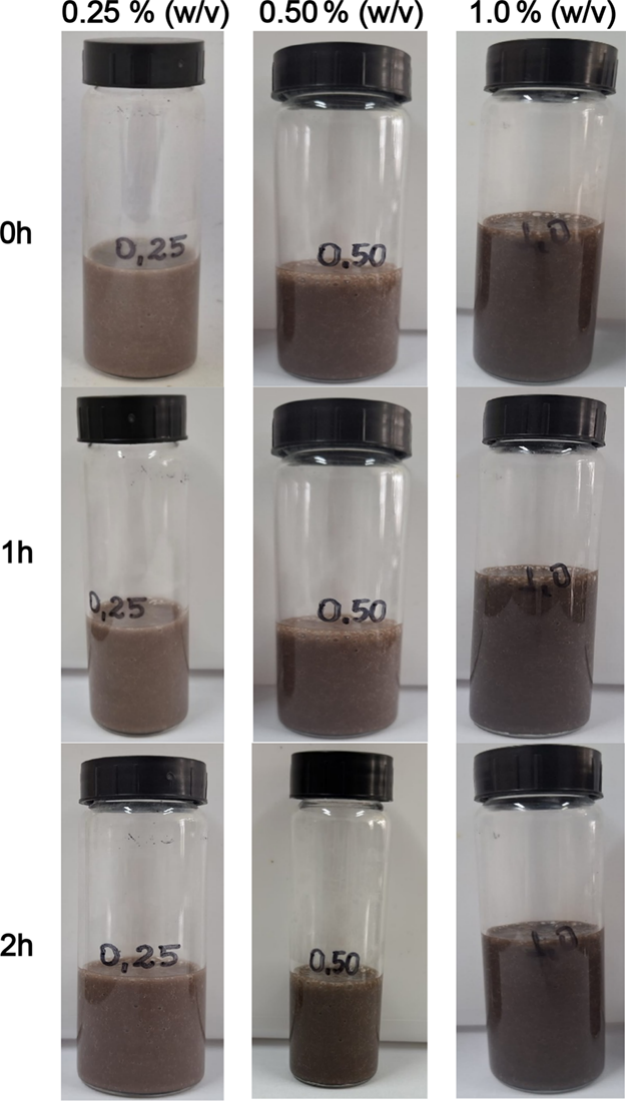
Visual sedimentation test of Fe_3_O_4_ nanoparticle suspensions at concentrations of 0.25, 0.5, and 1.0% (w/v), photographed immediately after preparation (0 h), and after 1 and 2 h of rest under ambient conditions. From left to right, the vials correspond to increasing concentrations.

Based on the results in Table 3, nanoparticles in the drilling fluid altered the behavior of all parameters obtained in the return permeability tests compared to those without nanoparticles. The following section presents the correlation between parameters to investigate their possible relationships.

Figure 7 presents the graph correlating nanoparticle concentration and return permeability obtained from the tests.

**Figure 7:**
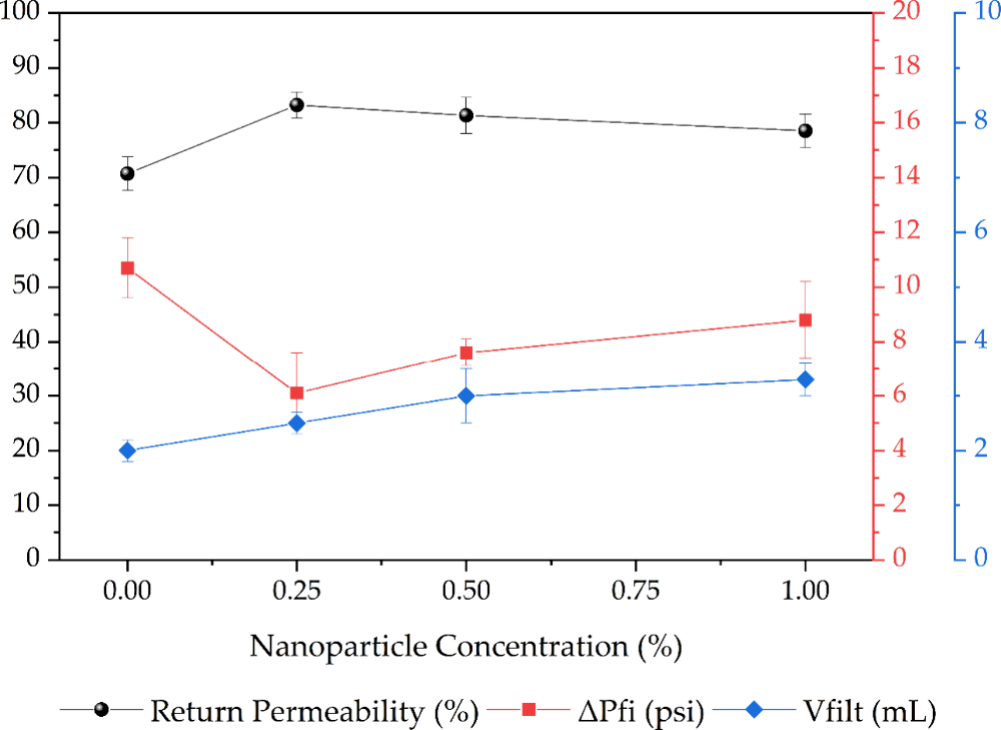
Curves of return permeability, flow initiation pressure, and filtrate volume as a function of nanoparticle concentration in the drilling fluid.

Based on the results obtained, it is observed that, in all return permeability tests involving nanoparticles in the drilling fluid formulation, the return permeabilities were higher than those in which the particles were not used. In this context, iron nanoparticles can create more robust and efficient bridges between the fluid components, resulting in an effective filter cake that minimizes solid invasion and, consequently, formation damage.^11^ Additionally, nanoparticles can alter the surface potential of the rock fines, reducing the repulsion between fines and grains, thereby preventing fines detachment and decreasing the likelihood of pore blockage and permeability reduction.^35^

Figure 8 presents a visual comparison of the drilling fluids and the surface of carbonate rock samples after testing, highlighting the differences in appearance and deposition between formulations with and without Fe_3_O_4_ nanoparticles.

**Figure 8:**
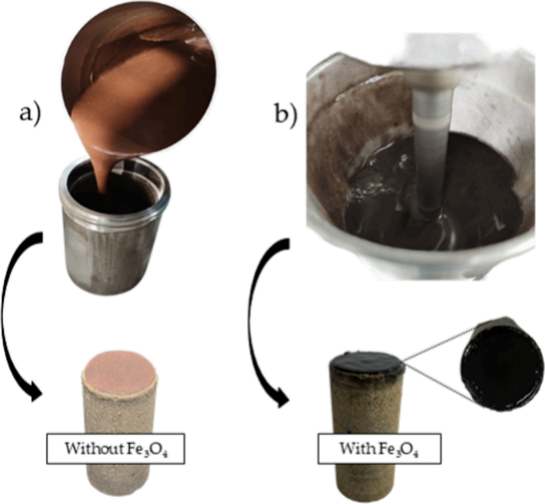
Drilling fluids and carbonate rock surfaces after return permeability tests: (a) without Fe_3_O_4_; (b) with Fe_3_O_4_ nanoparticles.

Another parameter that supports these observations is the flow initiation pressure (ΔPfi), or “cake lift-off pressure,” which represents the pressure required to initiate flow after the filter cake has formed. The tests in which nanoparticles were added to the fluid showed lower average values for this parameter (6.1–8.8 psi) than those without nanoparticles (10.7 psi).

Suri and Sharma^36^ concluded in their studies that the extent of solid invasion from the drilling fluid and the compressive strength of the internal filter cake are key factors controlling the flow initiation pressure. Indeed, when observing the behavior of ΔPfi and return permeability, an inverse relationship between these two parameters can be noted, indicating that the greater the formation damage (i.e., lower return permeability), the higher the pressure required to initiate brine production during flowback. It is believed that greater solid invasion occurred in the return permeability test using the drilling fluid without nanoparticles, resulting in a higher ΔPfi.

Regarding the filtrate volume, it was observed that the average filtrate volume increased as nanoparticles were added to the fluid. Some studies involving high-pressure filtration have shown that, up to a specific concentration of iron nanoparticles, the attractive and repulsive forces between the nanoparticles and the fluid components enable the formation of a filter cake in which a face-to-face layering dominates, reducing the available space for fluid invasion—that is, forming a filter cake with low porosity and permeability.^12,37^ In the tests conducted in this study, among the fluids containing nanoparticles, the 0.25% concentration resulted in the lowest filtrate volume, which increased gradually as more nanoparticles were added. This suggests that the higher degree of nanoparticle agglomeration may have destabilized the aggregation between the filter cake components and their effectiveness.

When comparing the results between filtrate volume and return permeability, it was expected that higher filtrate volumes would result in lower return permeabilities. However, when observing the average values from the tests without nanoparticles (0%), the opposite behavior was noted: the lowest filtrate volume (2.0 mL) and the lowest return permeability (70.7%) were obtained. In this case, fluid invasion caused by forming a filter cake composed of larger particles may have led to pore blockage near the core face, resulting in a small filtrate volume. However, due to the efficiency of this blockage, simple flowback was not sufficient to remove the damage, and consequently, lower return permeability values were obtained. The flow initiation pressure also supports this phenomenon, as higher pressures (10.7 psi) were required to overcome the blockage caused by the damage in that test. Although no direct microscopic analyses were performed in this study, the higher flow initiation pressure (10.7 psi) observed for the base fluid supports the hypothesis of severe pore blockage near the core face. This phenomenon, in which a compact filter cake composed of larger particles limits filtrate invasion but hinders permeability recovery, has been reported in previous studies.^12,37,38^ This limitation is acknowledged, and future work will include microscopic evaluation (e.g., SEM or micro-CT) to confirm the proposed mechanism. In contrast, although the filtrate volume was higher for the tests containing nanoparticles, the high surface-to-volume ratio of the particles makes them more easily removed by the standard production flow, thus increasing the return permeability.^38^ This ease of removal is confirmed by the lower flow initiation pressure values obtained in these tests.

Based on the results obtained, the Pearson correlation coefficient was used to assess the correlations between the different parameters (eq 5). This coefficient ranges from −1 to +1, where −1 indicates a strong negative correlation between two variables and +1 indicates a strong positive correlation. It is given by eq 5, where *n* is the total number of data points, *x̅* is the mean of the *x* values, *y̅* is the mean of the *y* values, *S_x_* is the standard deviation of the *x* values, and *S_y_* is the standard deviation of the *y* values.

$$r=\frac{1}{n-1}\sum \left(\frac{X-\mathrm{X̲}}{S_{x}}\right)\left(\frac{y-\mathrm{y̲}}{S_{y}}\right)$$ 5

Table 4 summarizes the results found among the parameters discussed in this section: nanoparticle concentration, return permeability, filtrate volume, and flow initiation pressure (ΔPfi).

**Table 4:** Pearson Correlation Coefficients between the Parameters Evaluated in the Tests with and without Nanoparticles^a^

		Pearson correlation	
parameter 1	parameter 2	all tests	tests with Fe_3_O_4_
nanoparticle concentration	RP	0.38	–0.99
	filtrate volume	0.96	0.94
	ΔPfi	–0.16	0.96
(RP)	filtrate volume	0.56	–0.97
	ΔPfi	–0.97	–0.98
filtrate volume	ΔPfi	–0.34	0.99

a (RP): return permeability.

A strong correlation was observed among all evaluated test parameters containing only nanoparticles (0.25, 0.5, and 1%). This behavior suggests that adding nanoparticles introduces a more systematic relationship between return permeability, filtrate volume, and flow initiation pressure (ΔPfi). Specifically, the strong negative correlation of −0.99 between nanoparticle concentration and return permeability indicates that increasing nanoparticle concentration is associated with a reduction in return permeability. This effect may be related to increased pore obstruction due to nanoparticle deposition, which impacts the flow capacity of the porous medium.^35^

Additionally, the strong negative correlation between return permeability and filtrate volume (−0.97) in the tests with nanoparticles reinforces the hypothesis that the formation of a nanoparticle layer on the pore surfaces reduces filtrate loss, but at the cost of greater flow restriction in the porous matrix. The high positive correlation between filtrate volume and ΔPfi (0.99) in the nanoparticle-containing tests indicates that a higher filtrate volume is associated with a greater pressure required to initiate flow, which may be related to the blocking effect caused by the accumulation of solid particles in the pore throats.

On the other hand, when considering all tests, including those without nanoparticles, the linearity of the correlations becomes less pronounced. It is noted that only two correlations remained strong: between nanoparticle concentration and filtrate volume (0.96), and between return permeability and ΔPfi (−0.97). This behavior suggests that, in the absence of nanoparticles, other flow and solid deposition mechanisms may influence the results, reducing the predictability of the observed relationships.

To support the interpretation of the return permeability results, additional statistical analyses were conducted. A one-way ANOVA revealed statistically significant differences among the groups (*p* = 0.0085), confirming that the nanoparticle concentration had a measurable effect on reducing formation damage. Post hoc analysis using the Tukey test revealed that the 0.25 and 0.50% Fe_3_O_4_ formulations yielded significantly higher return permeability values compared to the control group (*p* = 0.010 and *p* = 0.031, respectively). In contrast, the 1.0% group did not differ significantly from the control (*p* = 0.132). These findings reinforce that the 0.25% Fe_3_O_4_ formulation offers the most favorable balance between permeability recovery, formation protection, and additive dosage, suggesting that higher concentrations may not yield additional benefits and could even diminish performance due to potential aggregation or saturation effects.

The data indicate that adding nanoparticles significantly alters the system’s behavior, fostering more consistent interactions among the analyzed parameters. These findings underscore the benefits of using nanoparticles to minimize formation damage caused by the circulation of drilling fluids.

## 4 Conclusions

The present study investigated the influence of adding Fe_3_O_4_ nanoparticles to drilling fluids at concentrations of 0.25, 0.5, and 1.0% (w/v) and their ability to minimize formation damage in carbonate rocks. The fluid characterization indicated that incorporating nanoparticles did not significantly alter the fluid’s physicochemical and rheological properties, maintaining parameters such as viscosity, yield point, gel strength, pH, and relatively stable mud weight.

The return permeability flow tests revealed that nanoparticle fluids provided higher return permeability than conventional fluids. The fluid formulated with 0.25% Fe_3_O_4_ demonstrated the best performance, resulting in an increase in return permeability from 70.7 to 83.2% and a reduction in flow initiation pressure from 10.7 to 6.1 psi compared to the fluid without nanoparticles. However, adding nanoparticles also increased filtrate volume, suggesting a trade-off between filter cake formation and the fluid’s ability to minimize invasion into the porous matrix. The correlation analysis among the evaluated parameters indicated that nanoparticles directly influence the relationship between return permeability, filtrate volume, and flow initiation pressure.

The results highlight the potential of Fe_3_O_4_ nanoparticles as additives for optimizing drilling fluid formulations, reducing formation damage, and enhancing operational efficiency. However, the selection of nanoparticle concentration must be carefully considered, as higher concentrations may result in the formation of less efficient filter cakes and increased filtrate volumes. Further studies are suggested to assess the long-term stability of the nanoparticles and their interaction with various types of geological formations and to optimize their concentration according to the specific conditions of each reservoir.

Additional research may explore the use of nanoparticles beyond iron oxides and evaluate the particle size distribution of other solid components in the drilling fluid, as both factors likely influence filtration control and the mitigation of formation damage. These investigations would contribute to the further refinement of fluid formulations tailored to specific reservoir conditions.

In addition to the technical performance demonstrated, Fe_3_O_4_ nanoparticles present economic and environmental advantages. Magnetite is abundant and relatively low-cost compared with functionalized silica or carbon-based nanoparticles. Its magnetic properties enable potential recovery and reuse, reducing environmental impact and operational costs. Fe_3_O_4_ is also generally less toxic than other metallic oxides such as TiO_2_ and Al_2_O_3_, and its recycling potential enhances compatibility with sustainable drilling operations. Compared to silica nanoparticles, which are the most widely studied in drilling fluids, Fe_3_O_4_ offers stronger colloidal stability under alkaline conditions and the ability to form denser, thinner filter cakes in carbonate formations. These features make magnetite a promising and sustainable alternative for drilling fluid formulations.
